# Bcl-2△21 and Ac-DEVD-CHO Inhibit Death of Wheat Microspores

**DOI:** 10.3389/fpls.2016.01931

**Published:** 2016-12-26

**Authors:** Rakesh K. Sinha, Pavel Pospíšil, Priti Maheshwari, François Eudes

**Affiliations:** ^1^Cereal Biotechnology, Agriculture and Agri-Food Canada, LethbridgeAB, Canada; ^2^Department of Plant Anatomy and Cytology, Faculty of Biology and Environmental Protection, University of SilesiaKatowice, Poland; ^3^Department of Biophysics, Centre of the Region Haná for Biotechnological and Agricultural Research, Faculty of Science, Palacký UniversityOlomouc, Czechia

**Keywords:** cell death, microspore, Bcl-2△21, hydroxyl radicals, embryogenesis, caspase-3

## Abstract

Microspore cell death and low green plant production efficiency are an integral obstacle in the development of doubled haploid production in wheat. The aim of the current study was to determine the effect of anti-apoptotic recombinant human B-cell lymphoma-2 (Bcl-2△21) and caspase-3-inhibitor (Ac-DEVD-CHO) in microspore cell death in bread wheat cultivars AC Fielder and AC Andrew. Induction medium containing Bcl-2△21 and Ac-DEVD-CHO yielded a significantly higher number of viable microspores, embryo-like structures and total green plants in wheat cultivars AC Fielder and AC Andrew. Total peroxidase activity was lower in Bcl-2△21 treated microspore cultures at 96 h of treatment compared to control and Ac-DEVD-CHO. Electron paramagnetic resonance study of total microspore protein showed a different scavenging activity for Bcl-2△21 and Ac-DEVD-CHO. Bcl-2△21 scavenged approximately 50% hydroxyl radical (HO^•^) formed, whereas Ac-DEVD-CHO scavenged approximately 20% of HO^•^. Conversely, reduced caspase-3-like activities were detected in the presence of Bcl-2△21 and Ac-DEVD-CHO, supporting the involvement of Bcl-2△21 and Ac-DEVD-CHO in increasing microspore viability by reducing oxidative stress and caspase-3-like activity. Our results indicate that Bcl-2△21 and Ac-DEVD-CHO protects cells from cell death following different pathways. Bcl-2△21 prevents cell damage by detoxifying HO^•^ and suppressing caspase-3-like activity, while Ac-DEVD-CHO inhibits the cell death pathways by modulating caspase-like activity.

## Introduction

Isolated microspore culture (IMC) is of special research interest because this process produces homozygosity and is used as a platform for cost effective production of double haploid (DH) lines. IMC has been established in various crop species like barley (Kumlehn et al., 2006), triticale (Sinha and Eudes, 2015), oat (Sidhu and Davies, 2009), maize (Zheng et al., 2003), *Nicotiana* sp. (Garrido et al., 1995), wheat (Castillo et al., 2015), and *Brassica* sp. (Ferrie, 2003), for review see Ferri and Caswell (2011). In spite of a tremendous amount of effort, wheat IMC has not yet been shown to be efficient enough to be broadly implemented in wheat breeding programs. The rate of cell death of early to late uninucleate microspores, genotype dependency, low regeneration capacity of the produced embryo-like structure (ELS) and a low ratio of green vs. albino plants are the major limiting factors for IMC adoption in wheat breeding programs. The present study deals the problem of early microspores cell death, lower ELS production and low efficiency of green plant regeneration in wheat microspore culture.

Plants face a wide range of biotic and abiotic stresses throughout their lives and have employed a variety of defense strategies to overcome these stresses. One of the important features that plants utilize to attenuate these stresses is the formation of reactive oxygen species (ROS; Jaspers and Kangasjärvi, 2010; Sinha et al., 2012). The formation of ROS mostly takes place as a by-product of physiological processes in different intracellular compartments, like mitochondria or chloroplasts (Pospíšil, 2012). ROS are strong oxidizing agents which can oxidize and impair a large range of biological molecules (Gill and Tuteja, 2010). In fact, ROS play a dual role depending on their concentration and site of production. A lower concentration of ROS has been associated with developmental signals and the controlling of various aspects of plant biology (Gill and Tuteja, 2010), whereas a higher concentration of ROS has resulted in oxidative stress that may finally lead to cellular damage and programmed cell death (PCD; Gechev et al., 2006). The electron transport system of mitochondria has been considered as one of the important sources of electron leakage to oxygen, generating superoxide, which is then dismutated to H_2_O_2_. During the process a highly reactive HO^•^ is generated via Fenton and/or Haber-Weiss reactions, causing significant damage to cellular structure (Sharma et al., 2012). Earlier, it was shown that stress induced increases in NO production is associated with caspase induced PCD in barley microspore cultures (Rodríguez-Serrano et al., 2012) and defects in *N*-glycosylation induced caspase-like activity in yeast (Hauptmann et al., 2006). Although caspases play a major role as regulators of apoptosis in animal cells, plant genomes do not have similar caspase genes. Plant genomes do, however, encode proteinases like vacuolar processing enzyme (VPE). VPE has been shown to exhibit enzymatic properties similar to that of caspase 1 (Cai et al., 2014; Hatsugai et al., 2015). The molecular characterization of the VPE of Arabidopsis showed that βVPE is expressed in embryos (Kinoshita et al., 1995). VPE incites vacuolar rupture, initiating the proteolytic cascade leading to PCD in the plant immune response. VPE-dependent PCD pathway has shown to be involved in plant responses to a variety of stress and in the development of various tissues. VPE is proposed to control indirectly tonoplast rupture during PCD (Cai et al., 2014). The detailed mechanism by which VPE controls tonoplast rupture is still unclear. Besides the caspase-1 and caspase-3-like activities are involved in ER stress induced PCD in plants (Cai et al., 2014). Caspase-like activity has been shown in tobacco leaves infected with tobacco mosaic virus which specifically blocked by caspase-1 and caspase-3 inhibitors (Reape and McCabe, 2010). Likewise, caspase-3-like activity have detected in Arabidopsis protoplast following UV-C induced plant cell death (Wang et al., 2009), during the development of cow pea seed coat (Lima et al., 2015), in the barley pericarp (Tran et al., 2014) and caspase-3 inhibitor Ac-DEVD-CHO have tested to reduce cell death in embryonic callus in maize, barley, and triticale microspore suspension culture (Zheng et al., 2003; Rodríguez-Serrano et al., 2012; Żur et al., 2014; Lima et al., 2015; Sinha and Eudes, 2015). There are high possibilities that some of these proteolytic activities have been significantly enhanced upon induction of PCD. Therefore, there is a need to hunt for such plant ‘caspase-like’ proteases in future research.

The autolytic and non-autolytic are two main classes of PCD exist in plants (see review Van Doorn, 2011). However, autolytic PCD in xylem fibers was associated with increased expression of a mammalian BAG (Bcl-2-associated athanogene; Courtois-Moreau et al., 2009). It was reported that the plant BAG proteins regulate PCD during development and during pathogen attack (Doukhanina et al., 2006). Previously, OsNuc37, an endonuclease present in nuclei has shown to damage DNA during PCD in rice (Jiang et al., 2008). Earlier experiments have demonstrated that, the increased expression of *Bcl-2* in tobacco plants enhanced plant tolerance against different stresses (Chen and Dickman, 2004; Li and Dickman, 2004). Maraschin et al. (2003) have shown, for barley androgenesis, that the C-terminal proteolytic cleavage of the human isoform Bcl-2 caused death of non-embryogenic cultured microspores (Maraschin et al., 2003). However, embryogenic microspores do not show a decrease in viability (Maraschin et al., 2005). Bcl-2△21, truncated form of human Bcl-2, play an important role in antioxidant pathways to prevent cell death by protecting cells from H_2_O_2_ (Kamarehei and Yazdanparast, 2014). Recently Deng et al. (2015) have shown that Bcl-2 reduces G1 to S cell cycle transition in connection with a reduced ROS activity (Deng et al., 2015).

The present study was conducted to evaluate the effects of a caspase inhibitor on wheat microspore cell death and embryogenesis. We have transduced two different types of caspase inhibitor into wheat microspores, Bcl-2△21 and Ac-DEVD-CHO, and observed their potential action in improving microspore embryogenesis. Our results show that both Bcl-2△21 and Ac-DEVD-CHO were able to increase the cell viability, number of embryo-like structures, and total green plants regenerated in wheat. Both caspase inhibitors partially mitigated the formation of HO^•^, but the extent of mitigation was found to be different. Bcl-2△21 neutralized approximately 50% HO^•^ formed, whereas Ac-DEVD-CHO neutralized approximately 20% of HO^•^.

## Materials and Methods

### Growing Conditions and Collection of Donor Plant Material

Spring wheat cultivar AC Fielder and AC Andrew were grown in a growth cabinet with a photoperiod of 18 h per day (intensity 300 μE m^–2^ s^–1^), as mentioned in Sinha and Eudes (2015). The plants were treated with 2.5 ml l^–1^ Tilt^TM^ (propiconazole, Syngenta) before the tillering stage (Zadok et al., 1974) and Intercept^TM^ (0.004 g/L of soil, Imidacloprid, Bayer) once sufficient root development was established. The first seven tillers from each pot were harvested when microspores were in the mid to late uninucleate stage; the microspore stage was verified from a median floret using acetocarmine staining (Alexander, 1969). The remaining spikes were allowed to grow half their length out of the boot and then harvested for ovaries. Wheat spikes for microspore isolation were kept in the refrigerator (4°C) for 3 weeks with their bases in distilled water and their heads wrapped in aluminum foil. After 3 weeks ± 3 days, the spikes were extracted from the tillers and the most homogenous spikes were used for the microspore extraction.

### Microspore Isolation and Culture

Microspore culture was isolated as per Sinha and Eudes (2015). The homogeneous 10 spikes were collected and sterilized with 5.25% sodium hypochlorite for 3 min and then rinsed four times for 1 min with sterile distilled water with constant agitation. Anthers were aseptically dissected and placed in a sterile and refrigerated 110 ml waring blender cup (VWR International, #58983-093) containing 50 ml filter sterilized extraction buffer at 4°C. Anthers were then blended twice at speed of 18000 rpm for 7 s and the extract was filtered through a 100 μm sterile nylon mesh (VWR International, #CA21008-950) into two 50 ml centrifuge tubes (25 ml each). Further steps of microspores isolation and green plant regeneration were as Sinha and Eudes (2015). The number of independent replicates in each experiment is given in **Table 1**.

**Table 1:** Effect of Bcl-2△21 and Ac-DEVD-CHO treatment on embryo-like structure (ELS), green and albino plant regeneration in wheat.

	Mean (Fielder)				Mean (Andrew)			
Variable	*N*	ELS	GP	AP	*N*	ELS	GP	AP
Ac-DEVD-CHO	7	929a	10.4a	40a	4	508a	5.0a	24a
Bcl-2△21	7	910a	9.3a	39a	4	460b	4.5a	20a
Control	7	634b	6.1b	32a	4	367b	3.5a	26a

*N, represents the number of replicates; ELS, embryo-like structures, GP, green plants, and AP, albino plants. Values followed by the same letter do not differ significantly within each column (Tukey’s test, α ≤ 0.05).*

### Preparation of CPP-Protein Complex and Microspore Transduction

In the present study, a cell penetrating peptide (CPP) of plant origin was used to mediate protein delivery. This arginine rich CPP was identified by a 327 amino acid long *Oryza sativa* Japonica group (gene bank accession number AAN59771) and has the sequence RRRRRRRRHLRR (R_10_HL). Isolated microspores of Wheat (variety Fielder) were used as the plant material to test their protein delivery efficiency. The R_10_HL was synthesized *in vitro* (procured from Canpeptide Inc and Invitrogen) and tested for its protein delivery efficiency using the enzyme β-Glucuronidase (Sigma–Aldrich, *Escherichia coli* Type VII-A). Chariot (PAP1) protein delivering reagent was used as a positive control in the experiments.

Washed microspores after transduction were subjected to GUS histochemical staining using 5-bromo-4-chloro-3-indolyl glucuronide (X-Gluc) as the substrate. Transduced cells were then examined by visual inspection using a binocular compound microscope (**Figures 2d–f**). The percent transduction efficiency = Total no. of Blue cells/Total no. of cells × 100. The transduction efficiency of R_10_HL was 47.13%, while that of the chariot was 65.77%. Furthermore, the evaluation of the toxicity of R10HL and Chariot on microspore culture revealed that R10HL does not exhibit any toxicity up to a weight ratio of 1:6 (protein: R10HL), however, on the manufacturer recommended concentration Chariot exhibits toxicity. We have observed higher cell death after Chariot transduction compared to control and R_10_HL (data not shown). Bcl-2△21 and Ac-DEVD-CHO were complexed with R_10_HL in a weight ratio of 1:4 at room temperature for 15 min in phosphate buffer saline (PBS) (pH 7.4), and the control was R_10_HL-PBS. The complexed Bcl-2△21 and Ac-DEVD-CHO were transduced immediately after microspore isolation. Microspore (100,000) in 1.5 ml tubes were suspended in 150 μl of CIMC wash and were overlaid with the Bcl-2△21 or Ac-DEVD-CHO-R_10_HL complex to meet the final concentration of 500 nM for Human Bcl-2△21 (minus C-terminus; R&D Systems, USA) and 10 μM Ac-DEVD-CHO (Enzo life science, USA) and incubated at 28°C for 1 h with sporadic shaking. After incubation, 1 × 10^5^ microspores were transferred to each 50 mm Petri dish containing induction media. The final dose of Bcl-2△21 and Ac-DEVD-CHO in Petri dish was approximately 30 and 600 nM, respectively. The dishes were sealed with parafilm and placed in a 150 mm Petri dish around and an open 50 mm dish containing sterile distilled water in the middle of 150 mm Petri dish. Then the 150 mm Petri dishes were also sealed with parafilm and incubated in the dark at 28°C for 28 days. ELS were counted after 28 days using microscopy and recorded (**Table 1**).

### Flow Cytometry

Microspores were isolated from wheat spikes as mentioned above and the microspore concentration was determined using a haemocytometer. The microspore concentration was adjusted to 1 × 10^5^ cells per ml. The protein-R_10_HL complex was formed as mentioned above. The induction media without ficoll was used for incubation. After a specific time interval (24 h), 200 μL of homogenized microspores were collected for viability test. Fluorescein diacetate (FDA) was prepared as a stock solution in acetone at 1 mg/ml and immediately before use, was diluted 10-fold in wash buffer. The viability was determined by staining microspore with 0.01% FDA for 5 min in the dark at room temperature using a flow cytometer (Becton-Dickinson, San Jose, CA, USA). The percentage of dead (unstained by FDA) and live (stained by FDA) microspores was quantified for at least 10,000 microspores per treatment with fixed time points of 0, 24, 48, 72, 96, and 120 h. For statistical analysis, the data from at least three biological and at least two technical replicates were used, in which *n* = 10000 microspores.

### Labeling and Localization of Bcl-2△21

Protein Bcl-2△21 (2 mg/mL) was incubated in phosphate-buffered saline (PBS, pH 7.2, 100 mM bicarbonate) with the Alexa Fluor^®^ 647 (Enzo life science, USA), reactive dye having a succinimidyl ester moiety, at room temperature for 1 h with slow stirring. The unlabeled fluorophores were separated from the labeled protein solution using a resin column (provided in the kit) equilibrated previously with 1 × PBS elution buffer (0.1 M potassium phosphate, 1.5 M NaCl, pH 7.2, with 2 mM sodium azide). Protein concentration was evaluated according to a modified Bradford assay using the dye reagent from Bio-Rad and further confirmed by SDS-PAGE with subsequent Coomassie staining. Fresh microspore cultures were incubated for 1 h at 28°C in the dark, with 10 μM of labeled protein-R_10_HL complex in PBS (pH 7.2). For detection of viable cells, microspores were incubated with 10 μM FDA for 5 min. The samples were prepared and observed using confocal laser scanning microscopy (Olympus, FV1000) for imaging, as previously described by Sinha and Eudes (2015).

### Caspase Enzymatic Assays

Caspase-3-like activity was analyzed in IMC at time points 24 and 96 h, following the instruction of caspase-3 colorimetric assay kit (Enzo Life Sciences, Inc., Farmingdale, NY, USA). Duplicate microspore cultures were incubated with 500 nM of Bcl-2△21 and 10 μM of Ac-DEVD-CHO, as mentioned above. The reactions were mixed in the 96-well plates and incubated at 37°C for 2 h. Comparison of the absorbance (405 nm) of pNA from the treated sample with a control allows for the determination of the relative fold increase in caspase-3-activity. Fold increase in caspase activity was calculated using the following formula: (A405sample – A405 control)/A405 control. One relative unit is equal to onefold change. All assays were performed in five independent replicates and results were considered statistically significant when *p* < 0.05.

### Protein Isolation and Western Blot Analysis

For protein isolation, Bcl-2△21 (R&D Systems, USA, product details, 827-BC) treated and untreated microspores were collected at 24, 48, and 96 h and washed twice with wash buffer. Microspores were then treated with trypsin-EDTA (0.25% solution, Sigma–Aldrich) in 1:1 (v/v) ratio with wash buffer for 4 min at room temperature. The microspores were washed twice with wash buffer and pellets were collected in cryogenic storage vials (1 × 10^5^ cells/vial). The microspore pellets were first transferred to a liquid nitrogen tank and then stored in –80°C until use. Microspore pellets were homogenized using Precellys 24 Homogenizer equipped with a Cryolys cooling unit (Bertin Technologies) filled with six ceramic beads with a diameter of 1.4 mm (Bertin Technologies). The homogenized powder was suspended in 60 mM Tris, pH 6.8, 10% glycerol, 5% β-mercaptoethanol, and 2% SDS for total protein extraction. The protein extract was boiled for 10 min at 95°C and centrifuged twice at 15 000 ×*g* for 10 min to collect the supernatant. The concentration of total soluble protein was measured using Bradford method. Soluble proteins (5 μg) were separated on 12% Bis-Tris gels (Invitrogen, Carlsbad, CA, USA) and blotted onto nitrocellulose membranes. After removing the blot, membranes were first blocked for an hour through 5% milk dissolved in tris-buffered saline containing 0.05% Tween-20. The membrane was incubated with a 1:1000 dilution of Anti-His Tag HRP-conjugated Monoclonal Antibody (R&D Systems, USA) for an hour at room temperature. The blots were washed five times with distilled water, followed by enhanced chemiluminescence detection with Pierce ECL Western Blotting Substrate (Pierce, Rockford, IL, USA).

### EPR Spin-Trapping Spectroscopy

The presence of HO^•^ was detected by electron paramagnetic resonance (EPR) spin-trapping spectroscopy using DMPO (5,5-dimethyl-1-pyrrolin-*N*-oxide) as a spin-trap (Janzen, 1971). To determine the scavenging activity of the Bcl-2△21 and Ac-DEVD-CHO, total protein extracted from freshly isolated microspores was first mixed with either Bcl-2△21 or Ac-DEVD-CHO and in the presence of a 50 mM DMPO and 40 mM Mes (pH 7.2) then proteins were treated with 100 mM H_2_O_2_. After treatment, proteins were put in a glass capillary tube (Blaubrand^®^ intraMARK, Brand, Germany) and EPR spectra were recorded using an EPR spectrometer MiniScope MS400 (Magnettech GmbH, Berlin, Germany). The percent HO^•^ scavenging activity was calculated by the mean difference in the relative height of the central peak of the first derivative of the EPR absorption spectrum. The 100% reference value corresponds to the level of DMPO-OH adduct produced in the total protein in the presence of H_2_O_2_. The mean difference in DMPO-OH adducts with control and Bcl-2△21 and Ac-DEVD-CHO treated sample was calculated in percentage. EPR conditions were as in Sinha et al. (2012). A minimum of three independent replicates was used to calculate mean value in all EPR experiments.

### Assay for Peroxidase Activity

Peroxidase enzyme activities were determined as per the instructions provided in the assay manual (Sigma–Aldrich, USA). Microspore culture was incubated in multidisc plastic tray, each having 12-wells (Multidish, Nunclon Delta SI, Fa. Nunc, Denmark) in 2 mL induction media (without ficoll). After a fixed interval of time microspore were aliquoted into cryogenic storage vials (1 × 10^5^ cells/vial) and after centrifugation (150 ×*g* for 5 min), vials containing pellet were transferred to a liquid nitrogen tank and stored till used for further experiments. The homogenizations were performed with a Precellys 24 homogenizer equipped with a Cryolys cooling unit (Bertin Technologies) filled with six ceramic beads with a diameter of 1.4 mm (Bertin Technologies). The samples were homogenized for two intervals of 20 s at 5,500 rpm, with 2 min pause between each homogenization interval. After homogenization, the samples were suspended in an assay buffer and centrifuged for 10 min at 15,000 ×*g* to remove insoluble materials. Three technical replicates of each sample were made by transferring 50 μL samples to 96-well microplate. The fluorescence was measured using a Synergy MX Microtiter plate reader (Biotek Instruments, Inc.). The principles of the assay for peroxidase activity are based on the determination of the H_2_O_2_ decomposition resulting in a fluorometric (lex = 535/lem = 587 nm) product.

### Data Recording and Statistical Analysis

The development of the ELS was verified after 10–12 days of microspore culture incubation. The number of ELS was determined 27–30 days after isolation and subsequently, the structures were transferred to regeneration medium. The total number of regenerated green and albino plants was determined approximately 2–3 week after embryo transfer. The experiments were performed in a completely randomized design with a minimum of four replications, using spring wheat cultivar Fielder and Andrew. The resulting data were analyzed using the PROC GLM procedure of SAS for all variables studied (SAS, 2003). The least square means of the control treatment were compared with other treatments using REML methods in SAS. All values were expressed as the mean ± SD. A *p* value < 0.05 was considered to be statistically significant.

## Results

In the present study, we have analyzed the contribution of Bcl-2△21 and AC-DEVD-CHO, which are inhibitors of cell death in microspore culture. These inhibitors suppress caspase-3-activity, disarm HO^•^ and inhibit microspore cell death. We have demonstrated the effect of these proteins in preventing microspore cell death during the early stages of microspore culture and HO^•^ production; as well as the subsequent effects on production of green plants in wheat.

### Localization of Labeled Bcl-2△21 Protein

**FIGURE 1:**
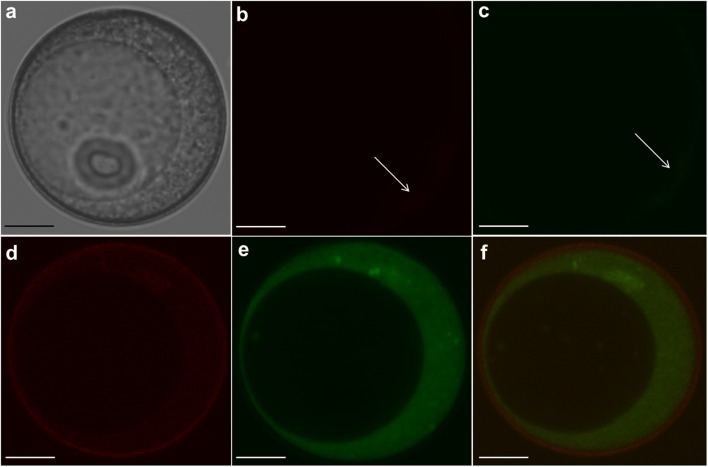
**Confocal microscopic localization of fluorescently labeled Bcl-2△21 in wheat isolated microspore.** Fluorescein diacetate (FDA) was used for microspores viability test and Alexa Fluor^®^ 647 labeled Bcl-2△21 (500 nM) was used to detect localization of microspores. Bars 20 μM. Green denotes FDA (aaa 488) and red fluorescence denotes Bcl-2△21 labeled Alexa Fluor^®^ 647. **(a)** Differential interference contrast microscopy (DIC) image; **(b)** autofluorescence of microspore in red channel; **(c)** autofluorescence of microspore in green channel; **(d)** Microspore treated with Bcl-2△21; **(e)** Microspore treated with FDA; **(f)** live microspore with combined red (exine) and green (cytoplasm) fluorescence. Arrow designates low autofluorescence signals in exine of microspore in the red and green channel.

Cellular localization of Bcl-2△21 was observed in viable FDA stained cells. Bcl-2△21 was labeled with Alexa Fluor^®^ 647 for tracking and observation in confocal microscopy. Small autofluorescence signals were detected in microspores exine in both red and green channels, but was not detected in the cytoplasm of microspores (**Figuers 1a–c**). Alexa Fluor^®^ 647 labeled Bcl-2△21 was mostly seen in the cytoplasm, but not in the vacuole (**Figure 1d**). The co-localization of both green and red fluorescent signals indicated the presence of Bcl-2△21 in the viable microspores (**Figure 1f**). The fluorescence of the FDA was brighter than the Alexa Fluor^®^ (**Figures 1d,f**), and a bright halo of red fluorescence was also systematically observed on the exine (**Figure 1**).

### Western Blot Analysis

**FIGURE 2:**
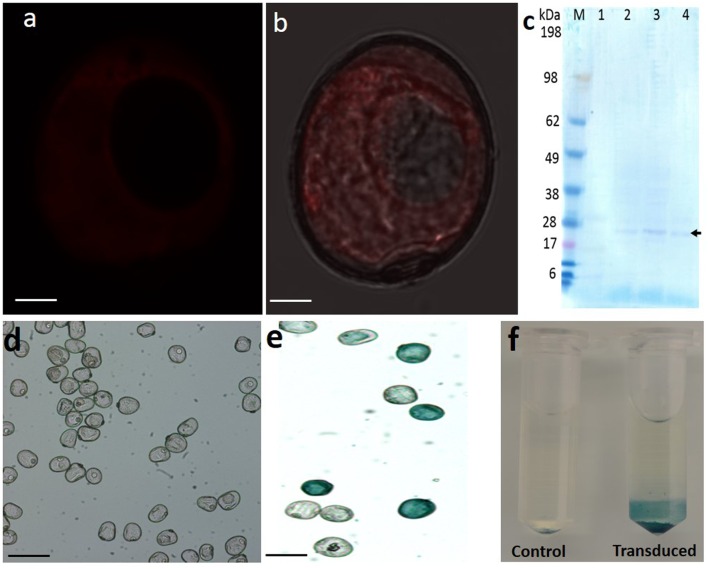
**Western blot analysis and GUS assay.** Total microspore protein were analyzed using western blotting with specific Anti-His HRP-conjugated antibodies (1:1000). **(a,b)** Confocal microscopy imaging of trypsin-EDTA (0.25%) washed microspores, red fluorescence denotes Bcl-2△21 labeled Alexa Fluor^®^ 647 inside the microspore but absent in exine. Bars: 20 μM. **(c)** Western blotting with specific Anti-His HRP-conjugated antibodies (1:1000). M, prestained benchmark protein marker (ThermoFisher Scientific). 1, total protein extracted from untreated microspores. 2–4, total protein extracted from Bcl-2△21 treated microspores. The arrow denotes the presence of specific Bcl-2△21 bands. GUS histochemical staining using 5-bromo-4-chloro-3-indolyl glucuronide (X-Gluc) as the substrate in microspores after transduction. **(d)** GUS protein without R10HL; **(e)** GUS protein conjugated with R10HL; **(f)** GUS assay without R10HL (control), GUS protein conjugated with R10HL (transduced). All other conditions were as mentioned in **Figure 1**.

The presence of Bcl-2△21 protein in the microspores was validated using Western blot analysis. After trypsin-EDTA treatment and washing, the presence of Bcl-2△21 labeled Alexa Fluor^®^ 647 inside the microspore was evaluated using Confocal microscopic imaging, which revealed the absence of Bcl-2△21 labeled Alexa Fluor^®^ 647 on the exine of microspore but present in the cytoplasm (**Figures 2a,b**). Total protein from Bcl-2△21 transduced microspores was isolated after 24, 72, and 96 h of microspores transduction and analyzed for the presence of Bcl-2△21. The presence of a faint band near 27–28 kDa protein marker has been identified in all Bcl-2△21 treated samples but not in control without treatment (**Figure 2c**).

### Effects of Bcl-2△21 and Ac-DEVD-CHO on Microspore Viability

**FIGURE 3:**
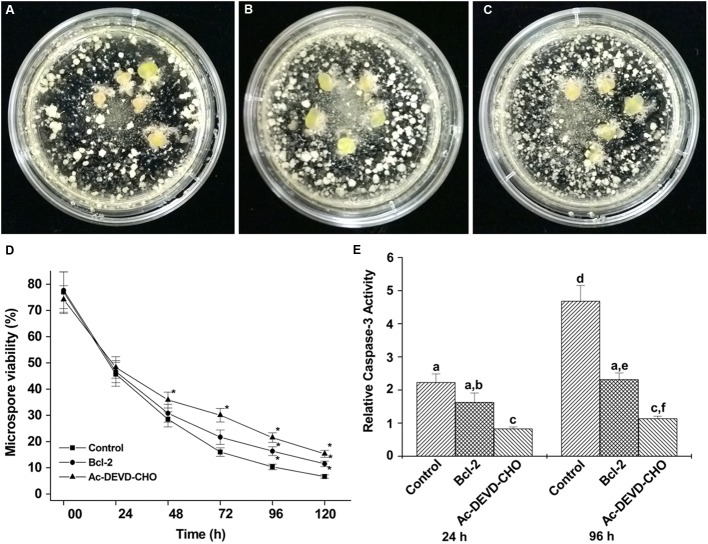
**The effect of Bcl-2△21 and Ac-DEVD-CHO on isolated microspore culture in wheat cultivar Fielder.** Bars 20 μM. **(A–C)** Isolated microspore culture after 4 weeks of inductive stress treatment and incubation, the figure showing large developing embryos; **(A)** control; **(B)** Bcl-2△21 and **(C)** Ac-DEVD-CHO treated microspore culture with supplemented ovaries; **(D)** the percent viability of microspore cells treated or not with Bcl-2△21 and Ac-DEVD-CHO; **(E)** comparison of caspase-3-like activity in control, Bcl-2△21 and Ac-DEVD-CHO treated microspore culture. The bars denote the mean standard error of at least five independent experiments. The asterisk (^∗^) and different letters indicate significant differences at *P* < 0.05 according to Tukey’s test.

The effect of Bcl-2△21 and Ac-DEVD-CHO on the early microspore cultures of wheat cultivar Fielder was observed at fixed intervals and compared to the control. The control microspore culture showed consistent reduction in viability, whereas the one treated with Ac-DEVD-CHO consistently showed higher cell viability starting at 48 h (**Figure 3D**). On the other hand, no significant difference in cell viability was observed in Bcl-2△21 treated cultures in the first 48 h compared to control cultures. The prominent difference in cell viability was observed during a period of 72–120 h of microspore culture. Cell viability after 96 h of incubation was significantly higher in both Bcl-2△21 (16.3%) and Ac-DEVD-CHO (21.5%) treated culture compared to the control (10.3%) (**Figure 3D**).

### Caspase-3-Like Activity and Microspore Cell Death

To examine the possible effect of caspases on microspore cell death, caspase-3-like activity was analyzed (**Figure 3E**) at two different experimental time points (24 and 96 h). As shown in **Figure 3E**, treatment of microspore cultures with Bcl-2△21 resulted in a reduction of caspase-3-like activity by 26% at 24 h and 50% at 96 h compared to control microspore cultures. The highest levels of caspase-3-like activity were detected in both 24 and 96 h control cultures (**Figure 3D**). Microspore cultures treated with Ac-DEVD-CHO resulted in significantly reduced levels of caspase-3-activities observed at both time points. The reduction in caspase-3-activities were observed in all time points when microspore cultures were treated with either Bcl-2△21 or caspase-3-inhibitor (Ac-DEVD-CHO; **Figure 3E**).

### Total Peroxidase Activity

**FIGURE 4:**
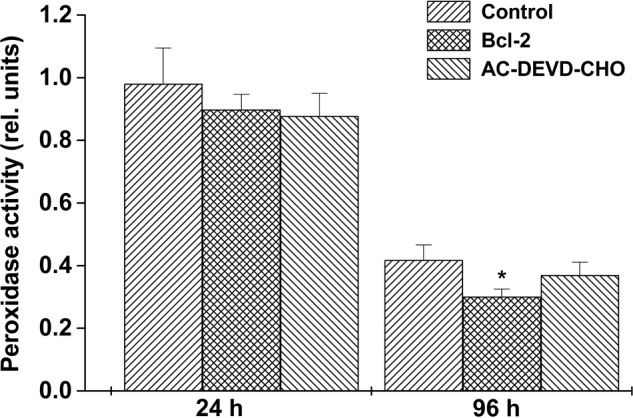
**The effect of Bcl-2△21 and Ac-DEVD-CHO on total peroxidase activities.** Total peroxidase activity was evaluated in wheat microspore suspension cultures in two different time points as described in “Materials and Methods.” The bars represent the standard deviation of the mean of five independent experiments. The asterisk (^∗^) indicates significant differences at *P* < 0.05 according to Tukey’s test. All other conditions were as mentioned in **Figure 1**.

Peroxidase activity was assayed in total protein extracts of control, Bcl-2△21 and Ac-DEVD-CHO treated wheat microspore cultures. After 24 h, peroxidase activity was observed to be similar in both control and treated microspore culture protein extracts. The highest peroxidase activity was observed in control protein extracts after 24 h of incubation. However, over a 4 days period the difference in the peroxidase activity between control and Bcl-2△21 treated microspore increased significantly (**Figure 4**). After 96 h of microspore culture a reduction in peroxidase activity was observed in the presence of Bcl-2△21 in comparison to Ac-DEVD-CHO and control.

### Effect of Bcl-2△21 and Ac-DEVD-CHO on HO^•^ Formation

**FIGURE 5:**
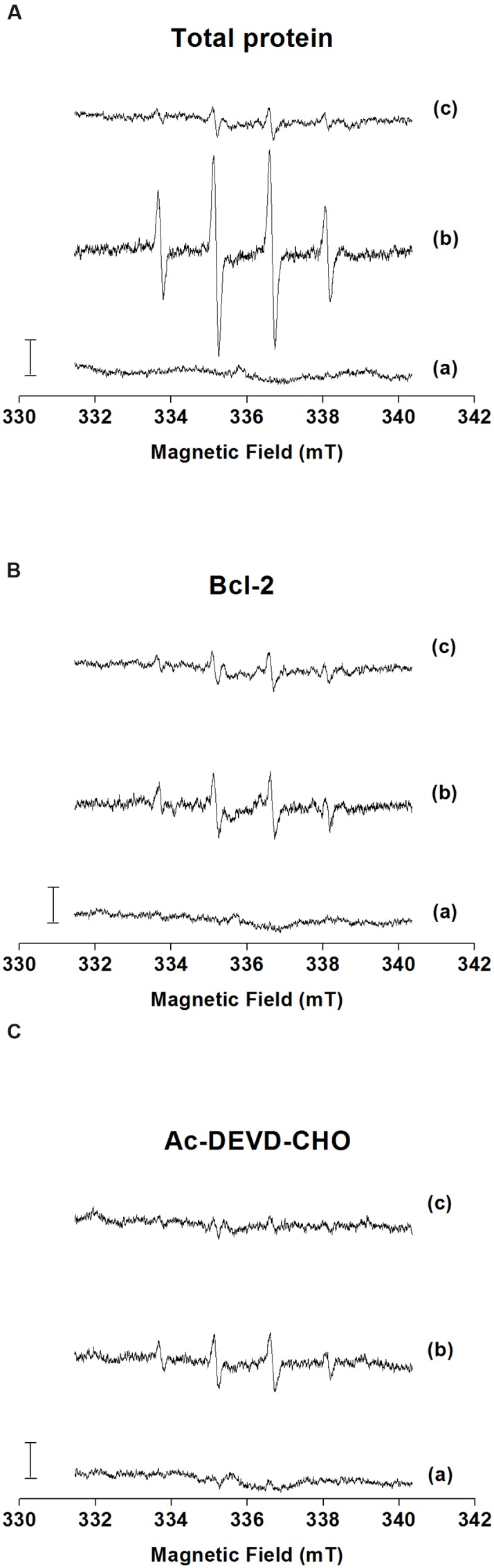
**Detection of DMPO-OH adduct electron paramagnetic resonance (EPR) signal in the presence of total protein, Bcl-2△21 and Ac-DEVD-CHO.** (Detection of HO^•^ formation in the presence of **(A)** total protein (5 μg) isolated from wheat microspore culture; **(B)** Bcl-2△21 (500 nM) and **(C)** Ac-DEVD-CHO (10 μM) using EPR spin-trapping spectroscopy. H_2_O_2_ (5 mM) was used to generate an EPR signal in each sample. H_2_O_2_-induced DMPO adduct spectra were measured in the presence of 50 mM DMPO and 40 mM Mes (pH 7.2). The intensity of EPR signal was evaluated as the relative height of the central peak of the first derivative of the EPR absorption spectrum. Letter **(a)** control, **(b)** H_2_O_2_, and **(c)** H_2_O_2_ + mannitol denotes trace a, trace b, and trace c, respectively.)

**FIGURE 6:**
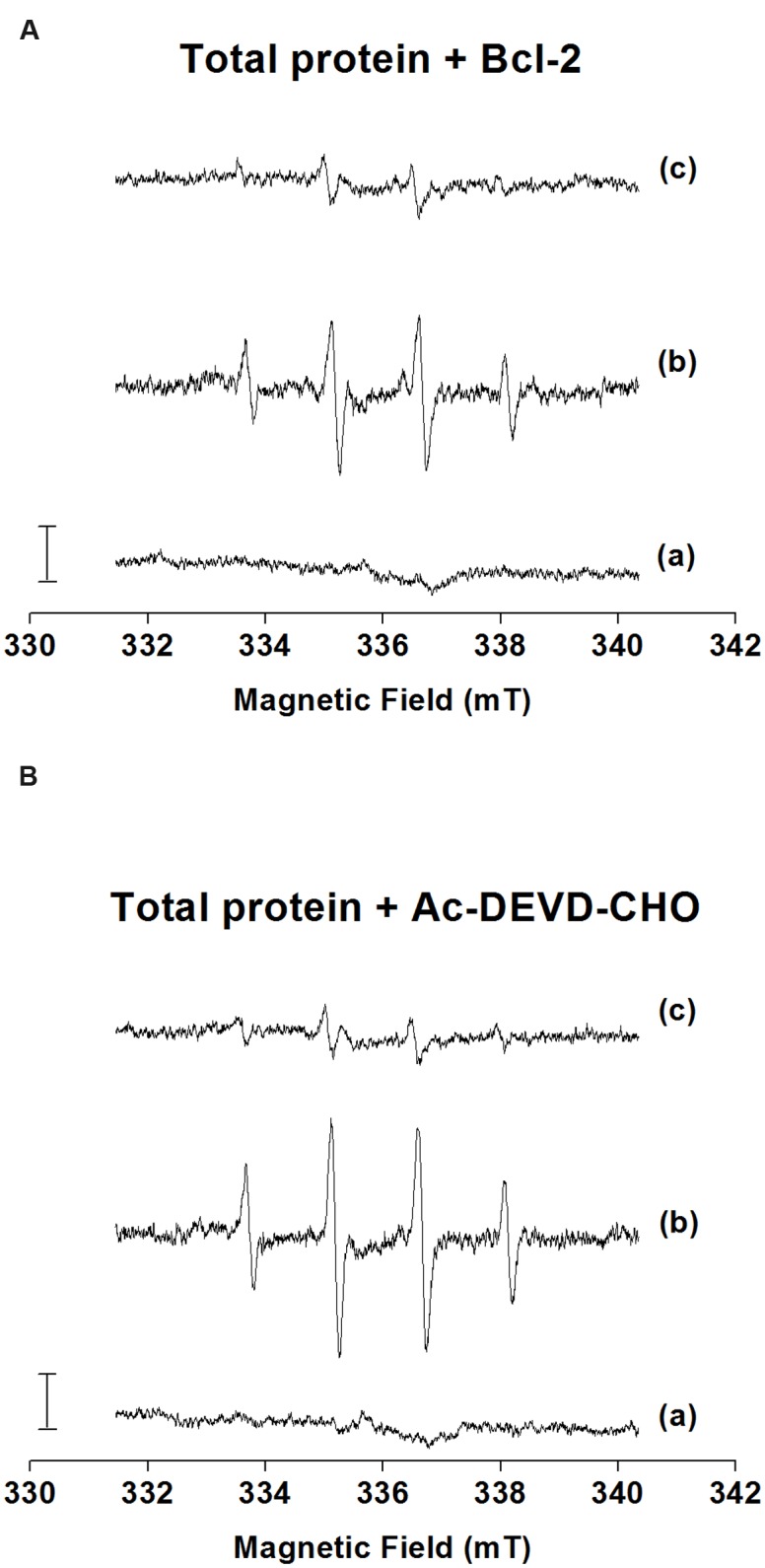
**Scavenging effect of Bcl-2△21 and Ac-DEVD-CHO on hydroxyl radical formation.** Detection of DMPO-OH adduct EPR signal in the total protein isolated from the wheat microspore culture in the presence of **(A)** Bcl-2△21 and **(B)** Ac-DEVD-CHO. H_2_O_2_-induced DMPO adduct spectra were measured in the presence of 50 mM DMPO and 40 mM Mes (pH 7.2). An amount of 5 ug of total protein was used for each measurement. Letter **(a)** control, **(b)** H_2_O_2_, and **(c)** H_2_O_2_ + mannitol denotes trace a, trace b, and trace c, respectively. All other conditions were as mentioned in **Figure 1**.

The formation of HO^•^ was measured after the addition of H_2_O_2_ to the total microspore protein, Bcl-2△21 and Ac-DEVD-CHO using EPR spin-trapping spectroscopy. The spin-trapping was accomplished in the presence of the spin-trap compound DMPO, which reacts with HO^•^ to form the DMPO-OH adduct. When DMPO was added to the total protein, Bcl-2△21 and Ac-DEVD-CHO, no DMPO-OH adduct EPR spectra were observed (**Figures 5A–C**, traces a). The addition of H_2_O_2_ to the total protein extract forms pronounced DMPO-OH signal (**Figure 5A**, trace b), while less pronounced DMPO-OH signal was observed after the addition of H_2_O_2_ to the Bcl-2△21 and Ac-DEVD-CHO (**Figures 5B,C**, traces b). When HO^•^ scavenger mannitol was added to the total protein, Bcl-2△21 and Ac-DEVD-CHO, DMPO-OH signal was almost completely suppressed (**Figures 5A–C**, traces c). These results indicate that HO^•^ was formed by reduction of H_2_O_2_ by metals via Fenton reaction. To explore antioxidant properties of Bcl-2△21 and Ac-DEVD-CHO, HO^•^ formation was measured in the total protein extract in the presence of Bcl-2△21 and Ac-DEVD-CHO. DMPO-OH signal decreased by 50% when Bcl-2△21 was added to the total protein (**Figures 5A** and **6A**, trace b; **Supplementary Figure S1**). Ac-DEVD-CHO resulted in a 20% decrease in DMPO-OH signal (**Figures 5A** and **6B**, trace b; **Supplementary Figure S1**). These observations revealed that Bcl-2△21 has the capability to eliminate HO^•^ formation after the addition of H_2_O_2_ to the total proteins.

### Effects of Bcl-2△21 and Ac-DEVD-CHO on Formation of Multicellular Structures

The growth pattern of Bcl-2△21 and Ac-DEVD-CHO treated microspores was frequently observed and found similar to the control. However, the number of ELS was different between Bcl-2△21 and Ac-DEVD-CHO treatments, and control (**Table 1**; **Figures 3A–C**). Bcl-2△21 showed 43% more ELS whereas Ac-DEVD-CHO showed 46% more ELS than the control.

## Discussion

Early microspore cell death and low green plant regeneration have been a recurrent challenge in the wheat microspore culture. It was not previously known whether the Bcl-2△21 and caspase 3 inhibitor Ac-DEVD-CHO could alter microspore developmental pathways. Here we report the use of anti-apoptosis Bcl-2△21 and caspase 3 inhibitor Ac-DEVD-CHO to disarm the production of HO^•^, thereby reducing cell death and increasing total green plant production, through their delivery into microspores. Our results indicate that Bcl-2△21 and Ac-DEVD-CHO protect cells from oxidative stress induced damage by disarming hydroxyl radicals and inhibiting the signal transduction pathways that regulates cell death. Bcl-2△21 protein, which lacks the C-terminal membrane-anchoring domain, has confirmed an anti-apoptotic effect, indicating that the anti-apoptotic mechanism was not dependent on the C-terminal of the molecule (Hockenbery et al., 1993). The Bcl-2△21-Alexa Fluor^®^ 647 fluorescence was visualized in the cytoplasm of microspores (**Figures 1d** and **2a,b**). In order to confirm the delivery of Bcl-2△21-Alexa Fluor^®^ 647 inside viable microspores, FDA was used during incubation (**Figures 1e,f**). This observation confirms the presence of Bcl-2△21-Alexa Fluor^®^ 647 specifically in the cytoplasm of the microspores. During complex formation, the CPPs forms a different type of non-covalent bonds with the cargo protein molecules (Bcl-2△21, Ac-DEVD-CHO), important ones including – Van der Waal forces, electrostatic interactions, and hydrophobic effects. Such type of bond formed is very quick and 15 min incubation is sufficient for the non-covalent complex formation (Chugh et al., 2009; Chang et al., 2014). Longer incubation leads to the bigger complexes eventually leading to the formation of aggregates. GUS protein without R10HL conjugation was not translocated into the microspores showed that it was R10HL leading to the internalization of the protein and help in translocation (**Figures 2b–e**).

The intensity of the western blot band was minimum at the 96 h post treatment sample and a maximum in the 48 h post treatment sample (**Figure 2c**). This represents the slow diffusion of Bcl-2△21 protein adhered to the surface of the microspores continued until 48 h. The presence of the Bcl-2△21 was exclusively observed in the cytoplasm and approximately absent in the vacuole (**Figures 2a,b**). However, the uptake of Bcl-2△21 protein into mitochondria and endoplasmic reticulum may have also been possible via a diffusion process. Such secondary diffusion may depend on the concentration of Bcl-2△21 inside the microspore. In mammalian cells, the function of Bcl-2△21 protein depends on their subcellular localization. Although many of the Bcl-2 family of proteins have been found in the membrane; including the mitochondrial outer membrane (MOM) and ER. There are approximately 60% Bcl-2 family soluble protein present in the cytosol and it has been proposed to translocate to the MOM during apoptosis (Wolter et al., 1997). Natural Bcl-2 is comprised of a carboxyl-terminal mitochondria targeting sequence, which is absent in the Bcl-2△21. However, due to lack of mitochondrial targeting sequence, Bcl-2△21 protein is mostly present in cytoplasm and maintains its ability to neutralize pro-apoptotic Bcl-2 family members and certain caspases (Gross et al., 1999), thereby protecting cells from cell death activation, cell injury, and the incidental death (Youle and Strasser, 2008). Relevant to this work, the abiotic stresses used in our studies are all known to generate ROS (Żur et al., 2014) and Bcl-2 inhibits ROS-induced cell death (Amstad et al., 2001; Jang and Surh, 2004).

Between both cultivars, Fielder was shown to respond better to microspore embryogenesis than AC Andrew. Both Bcl-2△21 and Ac-DEVD-CHO significantly increased the number of ELS in wheat cultivar Fielder (**Table 1**; **Figures 3A–C**). Furthermore, the ratio of green and albino plant regeneration was approximately similar in all the experiments, which nullify the direct involvement of Bcl-2△21 and Ac-DEVD-CHO in green plant regeneration. The significant increase in the total number of green plants/100.000 microspores was probably due to an increase in the total number of ELS/100.000 microspores.

The control wheat microspore culture showed the highest percentage of cell death (**Figure 3D**) as well as the highest caspase-3-like activity (**Figure 3E**). A clear shift toward higher microspore viability induced by Bcl-2△21 treatment occurred after 72 h (**Figure 3D**). This demonstrates that the effect of Bcl-2△21 was more prominent later than the effect of Ac-DEVD-CHO. The relative caspase-3-activity was significantly reduced in Bcl-2△21 and Ac-DEVD-CHO treated microspores at both 24 and 96 h time points. A correlation can be established between the reduced caspase-3-activity and the increased cell viability. Prior evidence suggests that several proteinases having caspase-like activity have been reported in plants (Xu and Zhang, 2009) and were involved in the regulation of plant cell death (Choi and Berges, 2013). Higher caspase-3-like activity has been associated with microspore cell death and has been reported after inductive stress in barley microspores and embryogenic suspension cultures (Rodríguez-Serrano et al., 2012) and in triticale microspore culture (Sinha and Eudes, 2015).

Bcl-2△21 treated microspores showed significantly less peroxidase activity compared to the control and Ac-DEVD-CHO at 96 h of microspore culture incubation (**Figure 3**). The increased level of total peroxidase is a defense process to protect cells against oxidative damage from H_2_O_2_ (Egea et al., 2007). Detoxification of H_2_O_2_ is a fundamental aspect of the cellular antioxidant response in which catalase and peroxidase have been used to decompose H_2_O_2_ in the presence of transition ions to a HO^•^ and hydroxyl ion (Pospíšil, 2012). In agreement with earlier results, this suggests that the presence of Bcl-2△21 down regulates the concentration of H_2_O_2_ (Li and Dickman, 2004; Luanpitpong et al., 2013).

The results of EPR studies reveal that Bcl-2△21 protein has the capability of retarding HO^•^ formation (**Figures 5** and **6**). It has been shown that H_2_O_2_ and other ROS are utilized in plant cell signaling to control different aspects of plant primary metabolism, cell defense, and the hypersensitive response (Foreman et al., 2003; Rentel and Knight, 2004). There is a wide range of possibilities that HO^•^ may be responsible for triggering the signaling pathways for microspore embryogenesis, and excess of this causes damage to the cultured cells. Bcl-2△21 mediated HO^•^ scavenging enhances microspore viability; reflecting that there is an excess of HO^•^ formation inside the microspores. However, Bcl-2△21 has been shown to block H_2_O_2_-mediated lipid peroxidation, and the over expression of Bcl-2△21 protects cells against oxidative damage in Jurkat T lymphoma cells (Kowaltowski and Fiskum, 2005). Several studies have produced similar findings that Bcl-2 prevents oxidative damage (Chen and Dickman, 2004; Li and Dickman, 2004). Żur et al. (2014) have observed an increased concentration of SOD in the anther of triticale DH responsive lines. The increased level of SOD help microspores against oxidative stress by dismutating superoxide anion radical to H_2_O_2_ and hence reducing the risk of the formation of the highly destructive oxidant, HO^•^ (Gill and Tuteja, 2010). Ac-DEVD-CHO scavenges small amounts of HO^•^, but also regulates the caspase activity in a higher range and hence reduces microspore cell death. This was in agreement with the prior results by Sinha and Eudes (2015). The contrasting results between Bcl-2△21 and Ac-DEVD-CHO treatments in HO^•^ scavenging and peroxidase activity suggested interplay among ROS formation, caspase activity, and cell death. This result also suggests a different mechanism of action for Bcl-2△21 and Ac-DEVD-CHO. Possibly, Bcl-2△21 plays a dual role, contributing to the regulation of cellular levels of ROS-induced intermediates (Hockenbery et al., 1993) and inhibiting the caspases-like activity associated with cytochrome c release (Zheng et al., 2015). While Ac-DEVD-CHO was mainly involved in the prevention of cell death by reducing caspase-3 activity.

## Conclusion

In summary, we report that Bcl-2△21 and Ac-DEVD-CHO complexed by R10HL can be taken into the plant cells, protect cells from oxidative damage and caspase-3-like activities by modulating the regulatory cell death pathways. Bcl-2△21 and Ac-DEVD-CHO reduce microspore cell death and produce increased numbers of ELS and have indirect effects in enhancing total green plant regeneration. Bcl-2△21 efficiently reduces cell death by detoxifying HO^•^ and inhibiting the caspase-3-like activity, whereas Ac-DEVD-CHO inhibits the cell death mostly by reducing caspase activity. Present results favor the concept that Bcl-2△21 functions upstream of caspase activation and prevents cell death by suppressing caspase-like activities (Renvoizé et al., 1998), while Ac-DEVD-CHO functions in direct inhibition of caspases. Both proteinaceous supplement efficiently led to increased ELS production efficiencies resulting in increased success of green haploid plant production.

## Author Contributions

RS carried out the IMC, confocal, flow cytometry, enzymatic activity experiments, participated in the data analysis and drafted the manuscript. PP carried out the EPR experiments and participated in the data analysis. PM provided the peptides for the experiments. FE participated in the design of the study, statistical analysis and helped to draft the manuscript. All authors read and approved the manuscript.

## Conflict of Interest Statement

The authors declare that the research was conducted in the absence of any commercial or financial relationships that could be construed as a potential conflict of interest.

## Supplementary Material

**FIGURE S1 | Hydroxyl radical scavenging by Bcl-2△21 and Ac-DEVD-CHO.** Effect of Bcl-2△21 and Ac-DEVD-CHO on the electron paramagnetic resonance (EPR) signal corresponding to the DMPO-OH adduct with 5,5-dimethylpyrroline-*N*-oxide (DMPO) was estimated. The 100% reference value corresponds to the level of DMPO-OH adduct produced in the total protein in the presence of H_2_O_2_. The mean difference in DMPO-OH adducts between control and Bcl-2△21 and Ac-DEVD-CHO treated sample was calculated in percentage. NA, represents with no addition; TP, total protein; DEVD, Ac-DEVD-CHO; Bcl-2, Bcl-2△21. Results are expressed as means ± SD values.

## References

[B1] Alexander M. P. (1969). Differential staining of aborted and non-aborted pollen. *Stain Technol.* 44 117–122. 10.3109/10520296909063335 4181665

[B2] Amstad P. A. Liu H. Ichimiya M. Berezesky I. K. Trump B. F. Buhimschi I. A. (2001). BCL-2 is involved in preventing oxidant-induced cell death and in decreasing oxygen radical production. *Redox Rep.* 6 351–362. 10.1179/135100001101536535 11865975

[B3] Cai Y. M. Yu J. Gallois P. (2014). Endoplasmic reticulum stress-induced PCD and caspase-like activities involved. *Front. Plant Sci.* 5:41 10.3389/fpls.2014.00041 PMC3924713 24592269

[B4] Castillo A. M. Sanchez-Diaz R. A. Valles M. P. (2015). Effect of ovary induction on bread wheat anther culture: ovary genotype and developmental stage and candidate gene association. *Front. Plant Sci.* 6:402 10.3389/fpls.2015.00402 PMC4471355 26150821

[B5] Chang M. Huang Y. W. Aronstam R. S. Lee H. J. (2014). Cellular delivery of noncovalently-associated macromolecules by cell-penetrating peptides. *Curr. Pharm. Biotechnol.* 15 267–275. 10.2174/1389201015666140617095415 24938892

[B6] Chen C. Dickman M. B. (2004). Bcl-2 family members localize to tobacco chloroplasts and inhibit programmed cell death induced by chloroplast-targeted herbicides. *J. Exp. Bot.* 55 2617–2623. 10.1093/jxb/erh275 15475374

[B7] Choi C. J. Berges J. A. (2013). New types of metacaspases in phytoplankton reveal diverse origins of cell death proteases. *Cell Death Dis.* 4:e490 10.1038/cddis.2013.21 PMC3734836 23412383

[B8] Chugh A. Amundsen E. Eudes F. (2009). Translocation of cell-penetrating peptides and delivery of their cargoes in triticale microspores. *Plant Cell Rep.* 28 801–810. 10.1007/s00299-009-0692-4 19288265

[B9] Courtois-Moreau C. L. Pesquet E. Sjödin A. Muñiz L. Bollhöner B. Kaneda M. (2009). A unique program for cell death in xylem fibers of *Populus* stem. *Plant J.* 58 260–274. 10.1111/j.1365-313X.2008.03777.x 19175765

[B10] Deng R. Hua X. Li J. Chi W. Zhang Z. Lu F. (2015). Oxidative stress markers induced by hyperosmolarity in primary human corneal epithelial cells. *PLoS ONE* 10:e0126561 10.1371/journal.pone.0126561 PMC4449087 26024535

[B11] Doukhanina E. V. Chen S. van der Zalm E. Godzik A. Reed J. Dickman M. B. (2006). Identification and functional characterization of the BAG protein family in *Arabidopsis thaliana*. *J. Biol. Chem.* 281 18793–18801. 10.1074/jbc.M511794200 16636050

[B12] Egea J. Rosa A. O. Cuadrado A. Garcia A. G. Lopez M. G. (2007). Nicotinic receptor activation by epibatidine induces heme oxygenase-1 and protects chromaffin cells against oxidative stress. *J. Neurochem.* 102 1842–1852. 10.1111/j.1471-4159.2007.04665.x 17540012

[B13] Ferri A. M. R. Caswell K. L. (2011). Isolated microspore culture techniques and recent progress for haploid and doubled haploid production. *Plant Cell Tissue Org. Cult.* 104 301–309. 10.1007/s11240-010-9800-y

[B14] Ferrie A. M. R. (2003). *Microspore Culture of Brassica Species*, 1st Edn. Berlin: Springer, 205–215.

[B15] Foreman J. Demidchik V. Bothwell J. H. F. Mylona P. Miedema H. Torres M. A. (2003). Reactive oxygen species produced by NADPH oxidase regulate plant cell growth. *Nature* 422 442–446. 10.1038/nature01485 12660786

[B16] Garrido D. Chibi F. Matilla A. (1995). Polyamines in the induction of *Nicotiana tabacum* pollen embryogenesis by starvation. *J. Plant Physiol.* 145 731–735. 10.1016/S0176-1617(11)81288-5

[B17] Gechev T. S. Van Breusegem F. Stone J. M. Denev I. Laloi C. (2006). Reactive oxygen species as signals that modulate plant stress responses and programmed cell death. *Bioessays* 28 1091–1101. 10.1002/bies.20493 17041898

[B18] Gill S. S. Tuteja N. (2010). Reactive oxygen species and antioxidant machinery in abiotic stress tolerance in crop plants. *Plant Physiol. Biochem.* 48 909–930. 10.1016/j.plaphy.2010.08.016 20870416

[B19] Gross A. McDonnell J. M. Korsmeyer S. J. (1999). BCL-2 family members and the mitochondria in apoptosis. *Genes Dev.* 13 1899–1911. 10.1101/gad.13.15.1899 10444588

[B20] Hatsugai N. Yamada K. Goto-Yamada S. Hara-Nishimura I. (2015). Vacuolar processing enzyme in plant programmed cell death. *Front. Plant Sci.* 6:234 10.3389/fpls.2015.00234 PMC4390986 25914711

[B21] Hauptmann P. Riel C. Kunz-Schughart L. A. Fröhlich K. U. Madeo F. Lehle L. (2006). Defects in N-glycosylation induce apoptosis in yeast. *Mol. Microbiol.* 59 765–778. 10.1111/j.1365-2958.2005.04981.x 16420350

[B22] Hockenbery D. M. Oltvai Z. N. Yin X. M. Milliman C. L. Korsmeyer S. J. (1993). Bcl-2 functions in an antioxidant pathway to prevent apoptosis. *Cell* 75 241–251. 10.1016/0092-8674(93)80066-N 7503812

[B23] Jang J. H. Surh Y. J. (2004). Bcl-2 attenuation of oxidative cell death is associated with up-regulation of gamma-glutamylcysteine ligase via constitutive NF-kappaB activation. *J. Biol. Chem.* 279 38779–38786. 10.1074/jbc.M406371200 15208316

[B24] Janzen E. G. (1971). Spin trapping. *Acc. Chem. Res.* 4 31–40. 10.1021/ar50037a005

[B25] Jaspers P. Kangasjärvi J. (2010). Reactive oxygen species in abiotic stress signalling. *Physiol. Plant.* 138 405–413. 10.1111/j.1399-3054.2009.01321.x 20028478

[B26] Jiang A. Cheng Y. Li J. Zhang W. (2008). A zinc-dependent nuclear endonuclease is responsible for DNA laddering during salt-induced programmed cell death in root tip cells of rice. *J. Plant Physiol.* 165 1134–1141. 10.1016/j.jplph.2007.12.008 18295371

[B27] Kamarehei M. Yazdanparast R. (2014). Modulation of notch signaling pathway to prevent H2O2/menadione-induced SK-N-MC cells death by EUK134. *Cell Mol. Neurobiol.* 34 1037–1045. 10.1007/s10571-014-0079-0 25005833 PMC11488918

[B28] Kinoshita T. Nishimura M. Hara-Nishimura I. (1995). Homologues of a vacuolar processing enzyme that are expressed in different organs in *Arabidopsis thaliana*. *Plant Mol. Biol.* 29 81–89. 10.1007/BF00019120 7579169

[B29] Kowaltowski A. J. Fiskum G. (2005). Redox mechanisms of cytoprotection by Bcl-2. *Antioxid. Redox Signal.* 7 508–514. 10.1089/ars.2005.7.508 15706098 PMC2570327

[B30] Kumlehn J. Serazetdinova L. Hensel G. Becker D. Loerz H. (2006). Genetic transformation of barley (*Hordeum vulgare* L.) via infection of androgenetic pollen cultures with *Agrobacterium tumefaciens*. *Plant Biotechnol. J.* 4 251–261. 10.1111/j.1467-7652.2005.00178.x 17177801

[B31] Li W. Dickman M. B. (2004). Abiotic stress induces apoptotic-like features in tobacco that is inhibited by expression of human Bcl-2. *Biotechnol. Lett.* 26 87–95. 10.1023/B:BILE.0000012896.76432.ba 15000473

[B32] Lima N. B. Trindade F. G. da Cunha M. Oliveira A. E. Topping J. Lindsey K. (2015). Programmed cell death during development of cowpea (*Vigna unguiculata* (L.) Walp.) seed coat. *Plant Cell Environ.* 38 718–728. 10.1111/pce.12432 25142352

[B33] Luanpitpong S. Chanvorachote P. Stehlik C. Tse W. Callery P. S. Wang L. (2013). Regulation of apoptosis by Bcl-2 cysteine oxidation in human lung epithelial cells. *Mol. Biol. Cell* 24 858–869. 10.1091/mbc.E12-10-0747 23363601 PMC3596255

[B34] Maraschin S. D. F. Lamers G. E. de Pater B. S. Spaink H. P. Wang M. (2003). 14-3-3 isoforms and pattern formation during barley microspore embryogenesis. *J. Exp. Bot.* 54 1033–1043. 10.1093/jxb/erg098 12598573

[B35] Maraschin Sde F Gaussand G. Pulido A. Olmedilla A. Lamers G. E. Korthout H. (2005). Programmed cell death during the transition from multicellular structures to globular embryos in barley androgenesis. *Planta* 221 459–470. 10.1007/s00425-004-1460-x 15645302

[B36] Pospíšil P. (2012). Molecular mechanisms of production and scavenging of reactive oxygen species by photosystem II. *Biochim. Biophys. Acta* 1817 218–231. 10.1016/j.bbabio.2011.05.017 21641332

[B37] Reape T. J. McCabe P. F. (2010). Apoptotic-like regulation of programmed cell death in plants. *Apoptosis* 15 249–256. 10.1007/s10495-009-0447-2 20094801

[B38] Rentel M. C. Knight M. R. (2004). Oxidative stress-induced calcium signaling in *Arabidopsis*. *Plant Physiol.* 135 1471–1479. 10.1104/pp.104.042663 15247375 PMC519063

[B39] Renvoizé C. Biola A. Pallardy M. Bréard J. (1998). Apoptosis: identification of dying cells. *Cell Biol. Toxicol.* 14 111–120. 10.1023/A:1007429904664 9553722

[B40] Rodríguez-Serrano M. Bárány I. Prem D. Coronado M. J. Risueño M. C. Testillano P. S. (2012). NO, ROS, and cell death associated with caspase-like activity increase in stress-induced microspore embryogenesis of barley. *J. Exp. Bot.* 63 2007–2024. 10.1093/jxb/err400 22197894 PMC3295391

[B41] SAS (2003). *Release 9.2.* Cary, NC: SAS Institute, Inc.

[B42] Sharma P. Jha A. B. Dubey R. S. Pessarakli M. (2012). Reactive oxygen species, oxidative damage, and antioxidative defense mechanism in plants under stressful conditions. *J. Bot. Artic.* 2012:217037 10.1016/j.plaphy.2016.05.038

[B43] Sidhu P. K. Davies P. A. (2009). Regeneration of fertile green plants from oat isolated microspore culture. *Plant Cell Rep.* 28 571–577. 10.1007/s00299-009-0684-4 19247663

[B44] Sinha R. K. Eudes F. (2015). Dimethyl tyrosine conjugated peptide prevents oxidative damage and death of triticale and wheat microspores. *Plant Cell Tissue Org. Cult.* 122 227–237. 10.1007/s11240-015-0763-x

[B45] Sinha R. K. Komenda J. Knoppova J. Sedlarova M. Pospisil P. (2012). Small CAB-like proteins prevent formation of singlet oxygen in the damaged photosystem II complex of the cyanobacterium *Synechocystis* sp. PCC 6803. *Plant Cell Environ.* 35 806–818. 10.1111/j.1365-3040.2011.02454.x 22070528

[B46] Tran V. Weier D. Radchuk R. Thiel J. Radchuk V. (2014). Caspase-like activities accompany programmed cell death events in developing barley grains. *PLoS ONE* 10:e109426 10.1371/journal.pone.0109426 PMC4186829 25286287

[B47] Van Doorn W. G. (2011). Classes of programmed cell death in plants, compared to those in animals. *J. Exp. Bot.* 62 4749–4761. 10.1093/jxb/err196 21778180

[B48] Wang C. L. Xu G. H. Jiang X. T. Chen G. Wu J. Wu H. Q. (2009). S-RNase triggers mitochondrial alteration and DNA degradation in the incompatible pollen tube of *Pyrus pyrifolia* in vitro. *Plant J.* 57 220–229. 10.1111/j.1365-313X.2008.03681.x 18786182

[B49] Wolter K. G. Hsu Y. T. Smith C. Nechushtan A. Xi X. G. Youle R. J. (1997). Movement of Bax from the cytosol to mitochondria during apoptosis. *J. Cell Biol.* 139 1281–1292. 10.1083/jcb.139.5.1281 9382873 PMC2140220

[B50] Xu Q. Zhang L. (2009). Plant caspase-like proteases in plant programmed cell death. *Plant Signal. Behav.* 4 902–904. 10.4161/psb.4.9.9531 19938376 PMC2802789

[B51] Youle R. J. Strasser A. (2008). The BCL-2 protein family: opposing activities that mediate cell death. *Nat. Rev. Mol. Cell Biol.* 9 47–59. 10.1038/nrm2308 18097445

[B52] Zadok J. C. Chang T. T. Konzak F. C. (1974). A decimal code for growth stages of cereals. *Weed Res.* 14 415–421. 10.1111/j.1365-3180.1974.tb01084.x

[B53] Zheng J. H. Viacava F. A. Kriwacki R. W. Moldoveanu T. (2015). Discoveries and controversies in BCL-2 protein-mediated apoptosis. *FEBS J.* 283 2690–2700. 10.1111/febs.13527 26411300

[B54] Zheng M. Y. Weng Y. Sahibzada R. Konzak C. F. (2003). “Isolated microspore culture in maize (Zea mays L.) production of doubled-haploids via induced androgenesis,” in *Doubled Haploid Production in Crop Plants*, eds Maluszynski M. K. Kasha J. Forster B. P. Szarejko I. (Berlin: Springer).

[B55] Żur I. Dubas E. Krzewska M. Janowiak F. Hura K. Ewa P. (2014). Antioxidant activity and ROS tolerance in triticale ( × *Triticosecale* Wittm.) anthers affect the efficiency of microspore embryogenesis. *Plant Cell Tissue Org. Cult.* 119 79–94. 10.1007/s11240-014-0515-3

